# Durability of anti-graffiti coatings on stone: natural vs accelerated weathering

**DOI:** 10.1371/journal.pone.0172347

**Published:** 2017-02-23

**Authors:** Paula M. Carmona-Quiroga, Robert M. J. Jacobs, Sagrario Martínez-Ramírez, Heather A. Viles

**Affiliations:** 1 School of Geography and the Environment, University of Oxford, Oxford, United Kingdom; 2 Department of Chemistry, Chemistry Research Laboratory, University of Oxford, Oxford, United Kingdom; 3 Departamento de Espectroscopía Nuclear, Vibracional y de Medios Desordenados, Instituto de Estructura de la Materia (IEM-CSIC), Madrid, Spain; Institute of Materials Research and Engineering, SINGAPORE

## Abstract

Extending the use of novel anti-graffiti coatings to built heritage could be of particular interest providing the treatments are efficient enough in facilitating graffiti removal and long-lasting to maintain their protective properties without interfering with the durability of the substrates. However, studies of the durability of these coatings are scarce and have been mainly carried out under accelerated weathering conditions, the most common practice for assessing the durability of materials but one that does not reproduce accurately natural working conditions. The present study aimed to assess the durability of the anti-graffiti protection afforded by two anti-graffiti treatments (a water dispersion of polyurethane with a perfluoropolyether backbone and a water based crystalline micro wax) on Portland limestone and Woodkirk sandstone after 1 year of outdoor exposure in the South of England with periodic painting and cleaning episodes taking place. A parallel study under artificial weathering conditions in a QUV chamber for 2000 hours was also carried out. Changes to the coatings were assessed by measuring colour, gloss, water-repellency, roughness and microstructure, the latter through micro-Raman and optical microscope observations, periodically during the experiments. The results show that both anti-graffiti treatments deteriorated under both artificial and natural weathering conditions. For the polyurethane based anti-graffiti treatment, artificial ageing produced more deterioration than 1 year of outdoor exposure in the south of England due to loss of adhesion from the stones, whereas for micro wax coating there were no substantial differences between the two types of weathering.

## Introduction

Coatings and stones have been linked from ancient times; from rock coatings that have contributed to the preservation of cultural assets by protecting the underlying surface of stones from weathering (patinas, heavy metals skins) or have enhanced their deterioration (soiling, sulfate crusts) [1] to current coatings formulations that are used in stone conservation [2] such as consolidants, water repellents, biocides, etc. Anti-graffiti coatings have been used for only the last 40 years, since the phenomenon of deliberate marking of surfaces with paints and pigments started, mainly on public transportation [3]. Recently, protective barriers that facilitate graffiti removal (with pressurized water spray and/or solvents) by generating low energy surfaces that make the substrate water- and oil-repellent have been applied to modern buildings [4]. Considering the high cleaning costs of graffiti removal (i.e. UK over £1billion a year [5] and USA $12 billion [6] development of effective anti-graffiti treatments is highly valuable. In fact, current advances of anti-graffiti coatings formulations with nanoparticles [7–9], powder technology, etc. for building retrofitting stand as proof of their increasing interest in the engineering field.

Graffiti also affects built heritage, but on historic materials the use of anti-graffiti treatments is still limited because of concerns about their impact on conservation. Commercial products on the market must satisfy demanding requirements: they should provide efficient protection with minimal modification of the historic substrate and without encouraging future damage.

Up to date, few studies have been made on such preventive treatments and their potential use on Cultural Heritage. Such treatments could be of particular value by reducing cleaning costs (especially when financial resources for conservation are still limited) and by improving the efficiency of graffiti removal, since traditional cleaning procedures are not always satisfactory and sometimes may damage the stone. Previous research has mainly focused on the compatibility principle which requires that the coatings do not significantly modify the physical properties of the stones such as colour, gloss, water absorption, water repellence, and water-vapour permeability, and on assessing the efficiency of graffiti removal on the protected surfaces after one or more cleaning cycles [3, 10–17]. Studies on the durability of these coatings are scarce and have been carried out under controlled laboratory rather than real-world conditions: (primarily) UV/condensation ageing [12,18–20], biodeterioration [3], salt crystallization [12] or frost resistance cycles [20]. An exception is the study by García and Malaga [12] which classified changes in the hydric properties and colour of 5 stones and 8 coatings after 10 months of exposure in Berlin [21].

Even though testing surface treatments under natural weathering conditions is of significant relevance for evaluating their performance, and as stated by Doehne and Price [2] (p. 52) is the “true test”, it is a highly time-consuming process. For this reason, accelerated weathering tests are more common in practice for assessing the durability of materials. These tests provide useful information by simulating different accelerated environmental conditions, alone or combined, in chambers: solar or UV radiation, rain, humidity, temperature, pollutants, etc. However, they do not reproduce all of them accurately and can produce abnormal effects on the materials not shown under natural conditions. Non-linearities can be found, with for example, an increase in intensity of the different environmental factors not resulting in an increase in the rate of the weathering reactions to the same extent [22]. For these reasons, it is recommended to run natural and artificial weathering tests in parallel [23].

Although several anti-graffiti coatings are available on the market there is a lack of knowledge of their behaviour under real long-term working (natural) conditions. This means that their safe application to built heritage (sufficient protection without the deterioration of coatings and substrates) is not proven. The aim of this study is to test two such products, a permanent and a sacrificial coating, on two popular English heritage stones, Portland limestone and Woodkirk sandstone. The approach taken is to run parallel field and laboratory tests of the durability and performance of these products, with periodical cleaning graffiti episodes included. A year-long field exposure trial in the South of England, where the Oxford Rock Breakdown laboratory (University of Oxford) is located, has been run. The temperate maritime climate of this area (Britain, Ireland and North-West Europe) is characterised by the absence of extreme temperatures and precipitations (the later are frequent but not extreme). In parallel, a laboratory test in an accelerated ageing QUV UV (ultraviolet) chamber (Q-Lab) was carried out. Properties such as colour, gloss, water-repellency (contact angle), roughness and microstructural modifications have been evaluated at intervals during the tests.

Whilst the research involves both natural and accelerated weathering tests it is not intended to establish a correlation between them because conditions in the chamber were not designed to replicate outdoor conditions and even if they were alike, geographical climatic variations, local weather variations, etc. would hamper such a comparative analysis [24].

## Experimental

### Coatings and stones

Two commercial anti-graffiti coatings were selected but this study can be replicated with similar coatings available in the market: a permanent product designed to withstand more than one cleaning cycle, and a sacrificial coating which is removed together with graffiti and thus would need to be reapplied after a cleaning procedure. The permanent coating is a water dispersion of polyurethane with a perfluoropolyether backbone (AG1) and the sacrificial coating is water based crystalline micro wax (AG2).

Both coatings were applied on three replicates each of four different slab sizes (150x75x10 mm, 85x65x10 mm and 75x35x10 mm and 30x25x10 mm) of Portland limestone and Woodkirk sandstone (APS Masonry, Oxford, UK). Size of the slabs was selected taking into account the requirements of the tests and space constraints in the UV chamber (Table 1). The stones, characterised both for their good durability, are commonly used in English built heritage. Portland limestone, quarried on the Island of Portland (Dorset, UK), has been extensively used in the South of England (St Paul´s Cathedral (London) and Buckingham Palace (London)) and around the world (United Nations headquarters (New York) and has been the first stone fully recognised as "Global Heritage Stone Resource" [25]. Woodkirk sandstone, quarried in Leeds (UK) has been extensively used since the 18^th^ century in paving and buildings in many cities in the UK [26].

**Table 1:** List of tests and slab sizes.

	Without graffiti application			After cleaning graffiti	
	UW (T/UT)	AW (T/UT)	NW (T/UT)	UW,NW(T)	AW(T)
colour	• 75x35x10 (3)	75x35x10 (3)	85x65x10 (5)	150x75x10 (4*)	150x75x10 (4*)
	• 85x65x10 (5)				
gloss	• 75x35x10 (2)	75x35x10 (2)	85x65x10 (3)		
	• 85x65x10 (3)				
roughness	150x75x10 (4)			150x75x10 (4*)	150x75x10 (4*)
contact angle	30x25x10 (3)	75x35x10 (3)	30x25x10 (3)		
vapour perrmeability	75x35x10 (1)				
porosity	85x65x10 (1)				

Treated (T) and untreated (UT) samples, in triplicates, before (UW) and after artificial (AW) and natural (NA) weathering trials, and after cleaning graffiti at different time intervals. Dimensions of the samples in mm and in parenthesis the number of measurements per sample (or half-sample*).

Two coats of the treatments were sprayed (HVLP; High Volume Low Pressure) at room temperature on one face of the samples on consecutive days and the excess was brushed with a roller. The application procedure was based on previous trials bearing in mind available manufacturer´s instructions. Only for the porosity test the coatings were applied on all the faces of the specimens. The active residue from the coatings was calculated by drying samples at ambient temperature until constant weight was reached before and after application. Portland limestone (Jordan’s whit bed, Jurassic, UK) is a white oolitic limestone with low proportion of micritic matrix and scattered shell fragments. Oolites size ranges from 0.1 to 0.5 mm in diameter and shell fragments are around 5 mm.

Woodkirk sandstone (Carboniferous, UK) is a light brownish-buff, fine grained sandstone mainly composed of quartz with sericitization of feldspars and scattered mica crystals and opaque minerals (iron oxides).

### Weathering tests

Coated and uncoated samples of 150x75x10 mm and 75x35x10 mm were artificially weathered in a QUV chamber (Q-Lab Corporation) with UVB radiation (0.45 W/m^2^ at 313 nm) and condensation cycles for 2000 h following the artificial weathering conditions defined in method C of the ISO 16474–3 [27]. Because of the layout of the chamber, the area irradiated on the big slabs was 95 x 63 mm. Each cycle consisted of 4 h hours UV exposure at 60°C in dry conditions followed by 4 hours of condensation at 50°C with UV lamps off.

**Fig 1:**
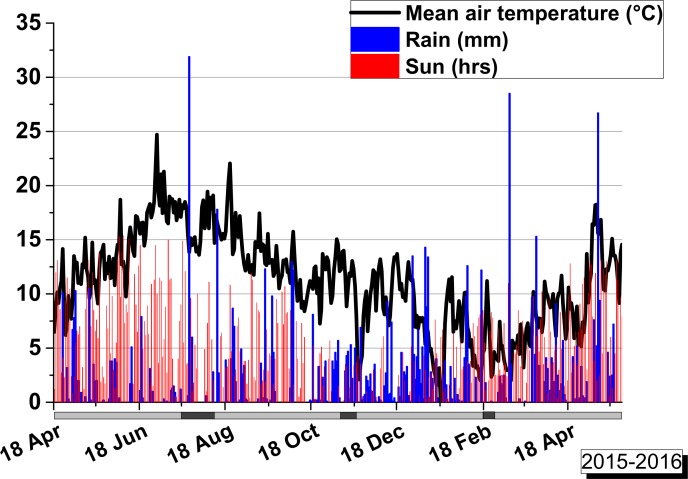
Climatic conditions for the outdoor exposure trial. Light grey bars represent the time in the field and the dark grey ones the time in the lab.

Specimens (150x75x10 mm, 85x65x10 mm and 30x25x10 mm) were naturally weathered in a temperate maritime climate in the South of England (Wytham Woods; 5 miles away from central Oxford in a non-polluted area) on a rack facing south for 12 months; from April 2015 to May 2016 (including time intervals in which samples were brought to the lab). Climatic conditions over the period were taken from the nearby Radcliffe Meteorological Station of the University of Oxford (Fig 1) and average solar radiation from the PVGIS Solar Radiation database [28] was 3070 Wh/m^2^ day. Over the year, the samples were exposed to a total of 1800 h of sun and 740 mm of rain, a range of temperatures between 0 and 25°C and UV radiation of 92.1 Wh/m^2^ day (3% of the average solar radiation) of which a 5% is accounted for UVB radiation (4.6 Wh/m^2^ day).

To summarize, samples in the chamber were exposed to a total of 1.6 MJ/m^2^ of UVB radiation, 1000 hours of condensed water and temperatures ranging from 50 to 60°C whereas in the outdoor exposure the environmental conditions were 6.0 MJ/m^2^ of UVB radiation, 740 mm of rain and temperatures between 0 and 25°C.

### Graffiti removal

In total, four painting and cleaning episodes were carried out for stones coated with AG1: consecutives in unweathered samples, after 3, 6, 9 and 12 months of outdoor exposure and after 500, 1000, 1500 and 2000 h in the QUV chamber. On the untreated samples, one cleaning cycle was also conducted. For the samples coated with AG2, only one painting-cleaning graffiti episode was carried out on the unweathered samples or at the end of both exposure times since as a sacrificial coating it is removed alongside graffiti.

A double layer of 5 spray paints (Madrid red, pistachio green, electric blue, black and silver from Montana Colors), sprayed from a distance of approximately 10 cm, and two layers of a black marker (water based pigment ink/tempera from Montana Colors) were applied on the surfaces of the slabs (150x75x10 mm) overlaid in a cross stripe pattern. Three long stripes were painted first (black marker, silver and black spray) continuing with six short stripes painted with the rest of paints 1 hour later (red, green and blue duplicated). For the artificially weathered samples, due to the small size of the irradiated area (95 x 63 mm), four short stripes instead six were painted with red, green and blue spray paints and an extra one with white (divinity white from Montana Colors).

Graffiti removal was performed 1 day after painting following recommendations that graffiti should be removed as quickly as possible to deter future attacks. For the AG1 and the untreated samples two cleaning procedures were followed (on each half of the 150x75x10 mm specimens): detergent (Dupli-Color Graffiti-Ex spray) and brush; detergent with high pressurized water spray (80 bars). Both procedures were done twice, after letting the detergent act on the surface each time for 15 minutes. Due to the small size of the irradiated area in the samples exposed in the QUV chamber, only the former cleaning method was carried out. For the sacrificial coatings high pressure hot water spray (110°C and 90 bars) was used following the coating manufacturer’s guidelines.

Efficiency in graffiti removal after each cleaning episode was evaluated through the determination of overall colour variations (ΔE*) on the painted areas of the surfaces of the stones with a Minolta CM-700d portable spectrophotometer. Ten measurements were taken on the 150x75x10 mm specimens with a stencil (Table 1). Total colour changes were determined (ΔE*) from the following equation (ΔE* = (ΔL*2+Δa*2+Δb*2)^1/2^); L* values measure lightness; a* measures the red (+)/green(-) hue and b* denotes the yellow (+)/blue (-) hue). Moreover, the aggressiveness of the cleaning methods was assessed by measuring the roughness of the surfaces after the final cleaning graffiti episode. The roughness parameter Rz (average distance between the tallest peak and the lowest valley in five sampling cut-offs) was measured with a Surtronic S128 roughness tester (stylus profilometer from Taylor Hobson; 5 measurements per area of interest, transect length of 2.5 cm, 400 μm range and 50 nm of resolution) on the 150x75x10 mm specimens (Table 1). The efficiency of the removal of the graffiti was also evaluated with stereographic microscope images (Leica MZ10 F Stereomicroscope).

### Physical properties

To assess the impact of the anti-graffiti treatments on physical properties of the stones, water-accessible porosity and water vapour permeability (δ), colour, gloss, contact angle and roughness were determined before and after the application of the anti-graffiti coatings (per triplicate) (Table 1). For the former property EN 1936:2006 standard [29] was followed (85x65x10 mm) and for the water vapour permeability the EN 1015–19:1999 standard [30] (75x35x10 mm) was employed. Colour was measured with the spectrophotometer mentioned above using the L*a*b* colour coordinates; five (85x65x10 mm) and three measurements (75x35x10 mm) per slab for the natural and artificial weathered samples respectively. Gloss measurements were taken at a specular reflection angle of 85°C with a TQC glossmeter (3 (85x65x10 mm) and 2 (75x35x10 mm) measurements per slab for the natural and artificial weathered samples respectively. The water repellence of the surfaces was evaluated with an IT Concept Tracker. Three drops of 5 micro litres of Millipore MilliQ water were deposited manually at 1mm/second onto the surface of the materials and the contact angle was measured from the theoretical drop shape found by using the Laplace-Young equation to fit the measured drop profile. Roughness parameter Rz was also determined as explained above on the 150x75x10 mm specimens.

To characterise the ageing of the coatings, colour, gloss and contact angle were determined also after the different weathering times as described above (Table 1). Moreover, micro-Raman spectra were obtained from the surfaces of the materials before and after 500, 1000, 1500 and 2000 hours in the QUV chamber with a confocal Raman microscope Renishaw Invia equipped with a Leica microscope and an electrically refrigerated CCD camera. Laser excitation lines were provided by a diode laser (785 nm) and a Renishaw Nd;YAG laser (532 nm) to explore the presence of the coatings respectively on limestone and sandstone. The laser beam (2.5–5 mW power at 785 nm, 0.5–2.5 mW at 532 nm) was focused over the sample with x50 magnifications. Typical spectra from 100 to 4000 cm^-1^ were recorded with a resolution of 4 cm^-1^. The time acquisition was 10 s and 1–5 scans were recorded to improve the signal-to-noise ratio. The frequencies were calibrated with silicon.

Statistical analysis (One way analysis of variance) was carried out where appropriate with SigmaPlot software (pairwise multiple comparison).

## Results and discussion

### Compatibility

Table 2 lists the physical properties of the two substrates before and after being treated with the two anti-graffiti coatings. More active residue remained on the stones coated with the permanent product (AG1) than on those treated with the sacrificial coating (AG2) accompanied by a greater decrease in their surface roughness (Rz).

**Table 2:** Physical properties of the stones before and after being coated with the permanent (AG1) and sacrificial (AG2) anti-graffiti coating.

	Portland LIMESTONE			Woodkirk SANDSTONE		
	Untreated	AG1	AG2	Untreated	AG1	AG2
Residue (g/m^2^)	-	112.3 ± 9.9	22.2 ± 4.4	-	116.7 ± 9.3	61.3 ± 0.73
L*	75.54 ± 1.07	73.66 ± 0.64	73.45 ± 0.28	61.80 ± 0.48	58.79 ± 0.23	60.31 ± 0.40
a*	2.39 ± 0.16	2.79 ± 0.10	2.78 ± 0.04	2.41 ± 0.32	2.75 ± 0.16	2.36 ± 0.19
b*	10.62 ± 0.41	12.89 ± 0.39	12.93 ± 0.10	15.26 ± 0.62	16.66 ± 0.11	15.79 ± 0.03
ΔE*	-	4.12 ± 0.46	4.76 ± 0.17	-	3.91 ± 0.28	1.98 ± 0.07
Gloss at 85°	0.42 ± 0.05	1.34 ± 0.23	0.64 ± 0.08	0.30 ± 0	1.28 ± 0.44	0.41 ± 0.03
Roughness (Rz, μm)	132 ± 15	96 ± 17	128 ± 22	107 ± 11	64 ± 10	102 ± 10
contact angle	0	102.7 ± 4.9	87.1 ± 1.2	0	105.5 ± 1.6	101.4 ± 6.9
δx10^-12^ (kg/ m s Pa)	12.72 ± 2.24	3.01 ± 0.55	9.68 ± 0.77	10.67 ± 0.36	4.38 ± 0.13	8.73 ± 0.47
Porosity (%vol)	13.88 ± 0.38	14.19 ± 0.15	14.46 ± 0.47	8.25 ± 0.26	6.97 ± 1.12	8.0 ± 0.25

Means and standard deviations, N = 3 samples. δ = water vapour permeability.

Both anti-graffiti coatings made the surfaces water-repellent (contact angle ≥90°) with contact values around 100–105° (apart from AG2 on limestone, the more porous of the two materials, where the measured contact angle is slightly below 90° (87.1°)) without modifying the water accessible porosity of the substrates. One Way ANOVA shows that there are no statistically significant differences in porosity before and after application of the coatings (P = 0.222 for limestone and P = 0.125 for sandstone, α = 0.05). However, AG1 caused a drastic reduction in water vapour permeability, one of the most important properties to assess the suitability of protective coatings, of 76% in limestone and 59% on sandstone, whereas AG2 lowered the permeability by around 20% on both substrates. This major drawback of AG1 has been also reported by Maxová et al. [31] when testing anti-graffiti coatings based on solution of polyesters and dispersion of wax and by García et al. [11] with another polyurethane anti-graffiti product. These latter authors have found smaller decreases in the water vapour transmission rate of stones when applying sacrificial anti-graffiti coatings such as acrylate copolymers and paraffin polymers. However, in general the sacrificial coating tested in the present study (AG2) performed better than the aforementioned treatments by reducing the water vapour exchange by less than 25%, which is considered an acceptable value when assessing the performance of anti-graffiti coatings as defined by García and Malaga [12].

The coatings slightly darkened and yellowed the surfaces (L* decreased and b* increased), except for AG2 on sandstone. These variations are enough to be perceived by the human eye (ΔE* >3) but fully acceptable in conservation studies (ΔE*≤5, [32]). By contrast, García et al. [11] and Tarnowski et al. [3] have reported higher colour variations, the former after testing three of four anti-graffiti coatings on seven stones, an acrylate copolymer, a polyurethane and a ethyl methacrylate and the latter after applying a fluorinated polyuretehane (like AG1), a fluorosilane, and a silicone anti-graffiti coating on marble and sandstone. The slight increase of gloss on the surfaces treated with AG1 is not important since it is not visible to naked eye (<2; [12]).

### Durability

Durability of the coatings was evaluated by examining the coated surfaces of the materials with a stereographic microscope and micro-Raman spectroscopy and by recording changes in their colour, gloss and water-repellency at the different exposure times: 500, 1000, 1500 and 2000 hours for the artificially weathering test in the chamber and 3,6,9 and 12 months for the natural ageing.

**Fig 2:**
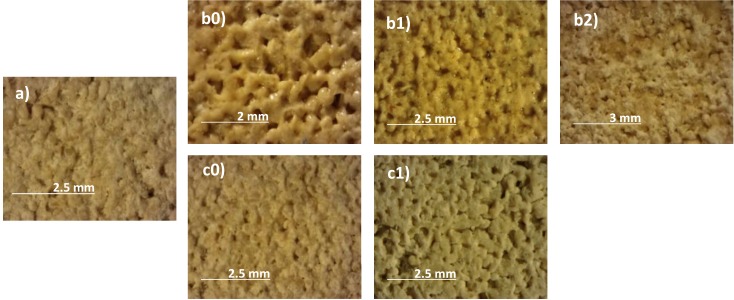
Stereographic microscopy images of the surface of limestone. Untreated (a) and coated with AG1 (b) and AG2 (c): unweathered (0), after 12 months of outdoor exposure (1) and after1500 hours (2) in a QUV chamber.

**Fig 3:**
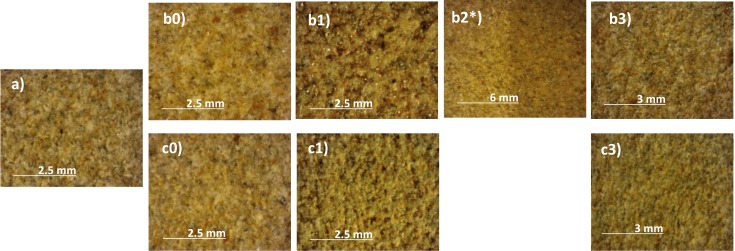
Stereographic microscopy images of the surface of sandstone. Untreated (a) and coated with AG1(b) and AG2(c): unweathered (0), after 12 months of outdoor exposure (1) and after 1500 (2) and 2000 hours (3) in a QUV chamber * Just the right half of the sample has been exposed to UVB radiation.

Following general visual inspection of the coated and uncoated materials with a stereographic microscope (Figs 2 and 3), only the AG1 anti-graffiti coating can be detected on both stones by leaving a glossy finish on their surfaces (Figs 2 and 3B0) whereas AG2 was unnoticed. However with the artificial weathering this glossy finish is lost on limestone and sandstone, but not after 1 year of outdoor exposure. This is opposite to Gagné´s findings [19] on polysaccharide anti-graffiti coatings whose gloss increased after artificial ageing with UV light and sprayed water. As can be seen in Fig 2, treatment is removed from the limestone surfaces (patchy effect) due to its loss of adhesion (Fig 2B2) while on sandstone the coating gets matte and dark (Fig 3B2*) and eventually removed.

**Fig 4:**
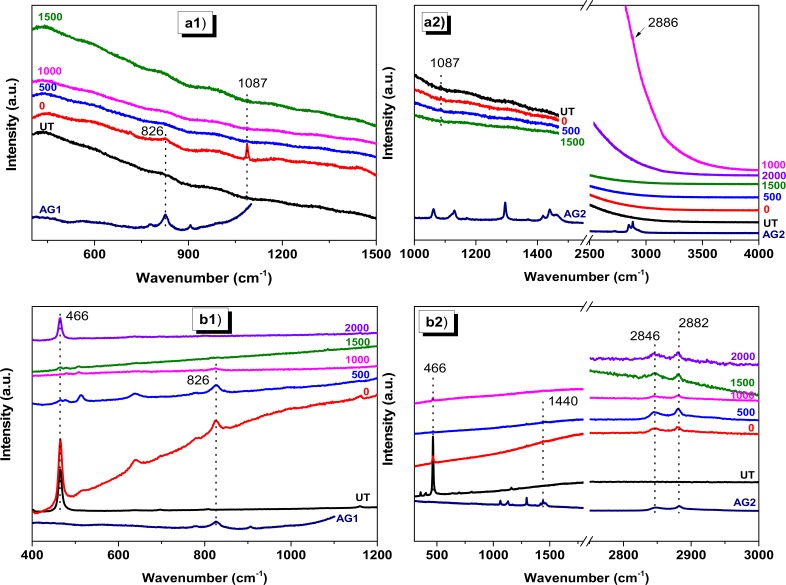
**Micro-Raman spectra of the coated surfaces of limestone (a) and sandstone (b)** AG1 (1) and AG2 (2) before (0) and after being weathered for 500, 1000, 1500 and 2000 h in a QUV chamber. Spectra of the uncoated surfaces (UT) and of the treatments (AG1 and AG2) are also shown as references.

The distribution of the coatings on the surface of the materials was also analysed by means of micro-Raman spectra obtained on the untreated, treated and artificially weathered samples (Fig 4). The band at 826 cm^-1^, generated by the symmetric stretching of C-O bonds of ether groups [33], was used to track AG1 (Fig 4A1 and 4B1). This band is visible up to 1000 h of weathering on sandstone spectra, where the band at 466 cm^-1^ belongs to quartz (Fig 4B1). On limestone the signal to track the coating was just observed on the unweathered surfaces, besides the band at 1087 cm^-1^ assigned to calcite (Fig 4A1). Nonetheless, due to the high fluorescence of limestone these results are not representative to clearly identify the presence or absence of the treatment as the fluorescence interferes with the readings [34].

AG2, that was undetected under the stereographic microscope, is clearly seen on the micro-Raman spectra of the artificially weathered sandstone at any given exposure time by tracking the vibration band of its alkyl groups, at 2846 and 2882 cm^-1^. Once again, on limestone the coating is difficult to detect (Fig 4A2 and 4B2).

**Fig 5:**
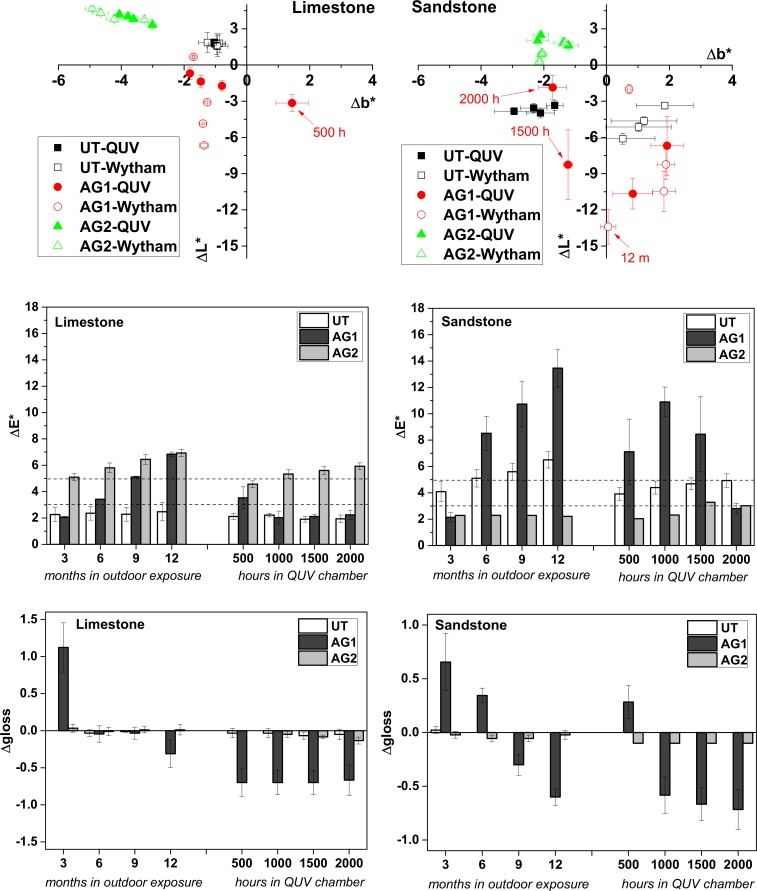
From top to bottom; Changes in colour coordinates, L* and b*, total colour variation (ΔE*) and gloss of limestone and sandstone. Untreated (UT) and anti-graffiti treated surfaces (AG1 and AG2), artificial (QUV) and natural weathering (Wytham) at different months of outdoor exposure or hours in the chamber.

Under artificial and natural weathering, AG2 experiences the same chromatic changes on both stone types by losing its yellow hue content (b* increases) and getting lighter (L* increases) (Fig 5A and 5B) AG1 coated surfaces on the contrary darkened, more on sandstone than in limestone, and yellowed just on the first degradation episodes. After 500 h in the QUV chamber the yellowing effect decreased or disappeared for the sandstone and limestone respectively, due to the progressive removal of the coating whereas for the naturally weathered samples yellowing is only observed on sandstone up to 9 months of outdoor exposure. It is worth noticing that uncoated sandstone gets dark, particularly under environmental conditions, because of the oxidation of their oxide minerals.

Regarding total colour variations (ΔE*) (Fig 5C and 5D), on sandstone the permanent anti-graffiti (AG1) is heavily weathered (ΔE* ranging from 7 to 14) under both types of weathering conditions from early on in both exposure times, 500 h and 6 months. Colour changes after 9 months of outdoor exposure are similar to the ones measured after 1000 h of UVB radiation in the QUV chamber. However under artificial conditions, 1500 and 2000 h, AG1 is removed from sandstone surfaces as revealed by stereographic microscopic examination (Fig 3B2 and 3B3), micro-Raman spectroscopy (Fig 4B1) and the decrease of ΔE* (Fig 5). On limestone, the light-coloured and more porous stone, colour changes on the surface treated with AG1 and weathered are less intense than in sandstone. In fact, the admissible threshold of overall colour of 5 units was exceeded just after 9 months of outdoor exposure (Fig 5). On the artificially weathered samples the colour changes recorded after the first 500 h of artificial weathering are below 5 units and decrease after 1000 h due to the removal of the coating as confirmed by microscopy. The poor performance of this polyurethane based anti-graffiti coating is in contrast with the traditional stability of these type of compounds towards UV light [21]. Nevertheless, Rabea et al. [18] have also found durability issues on a polyurethane anti-graffiti coating modified with silicone modified polyacrylate additives due to the deterioration of the latter under UV irradiation.

Colour changes of the surfaces coated with AG2 followed the opposite trend: they were more intense on limestone (ΔE* >5), particularly after outdoor exposure, than in sandstone (where they were inconspicuous (ΔE*<3)) for both types of weathering tests. These results are comparable with those from García [21] where a polymeric wax exhibited colour changes between 2.5 to 5.5 after a less intense artificial ageing trial (680 h in the chamber with UVA light). Gloss changes for both anti-graffiti were not prominent (Fig 5): since for AG1 were too small to be perceived by a human eye (<2 units) and for AG2 are practically 0 after the ageing trials.

**Fig 6:**
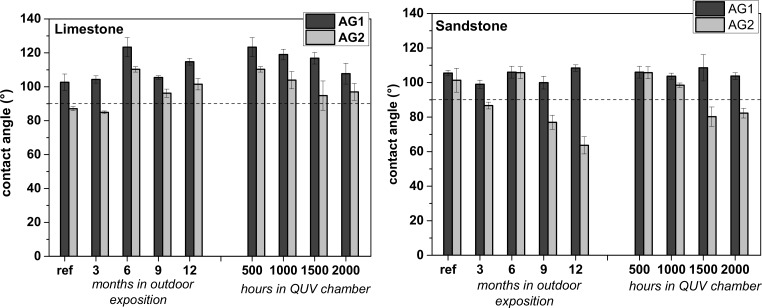
Contact angle of the anti-graffiti coated surfaces of limestone and sandstone after natural and artificial weathering.

On limestone, declines in the contact angle of surfaces coated with AG1 and AG2 after artificial ageing can be seen (Fig 6A), as reported by García [21] on stones treated with polyurethane and wax anti-graffiti coatings coating under UVA light and condensation, however the samples are still water repellent (>90°). On the contrary, for the naturally weathered samples where colour changes were higher, contact angles were not affected (results at 6 months are anomalous for both stones) because at least AG1 (unlike under artificial ageing) is not removed from the surface.

On sandstone (Fig 6), the contact angle of the AG2 coated surfaces gradually decreases under both weathering conditions making the surface no longer water repellent (<90°) even though colour changes were not perceptible to the naked eye (ΔE≤3) and the coating still remains on the surface after the artificial ageing (Micro-Raman analysis). On the contrary, despite of the heavy ageing of the AG1 coating on sandstone (high colour changes under both types of weathering) and of its removal from the surfaces after artificial ageing, the water repellency of the coated surfaces did not decrease. This would indicate that the removal of AG1 is not total.

To summarize, both anti-graffiti coatings deteriorated under natural and artificial exposure conditions. AG1 became heavily weathered as shown by the high colour variations and the artificial ageing produced more deterioration than 1 year of outdoor exposure since the coating is partially removed from the surfaces due to loss of adhesion. Weathering signs of AG2 coating are relevant colour changes on limestone and loss of water repellency (mainly) on sandstone. Regarding which ageing test is more aggressive there are no substantial differences. All pairwise multiple comparison procedures (Holm-Sidak method; One Way ANOVA) of ΔE* and contact angle at the end of both exposure times reveal that for colour, on limestone natural weathering is more damaging than artificial, whereas on sandstone artificial ageing is more damaging than natural (P<0.05). On the other hand, there are no significant differences when comparing contact angles measured at the start and end of each trial on both sandstone and limestone (P>0.05, α = 0.05).

### Cleaning efficiency

To assess the effectiveness of the graffiti removal on the unweathered and weathered surfaces, samples were visually inspected and colour coordinates were measured. Moreover, since two of the cleaning procedures involve the use of pressurized water spray (the only method recommended for the sacrificial coating) it is of the utmost importance to also evaluate any change in the roughness of the stones that can adversely facilitate the access of water, pollutants or future graffiti.

**Fig 7:**
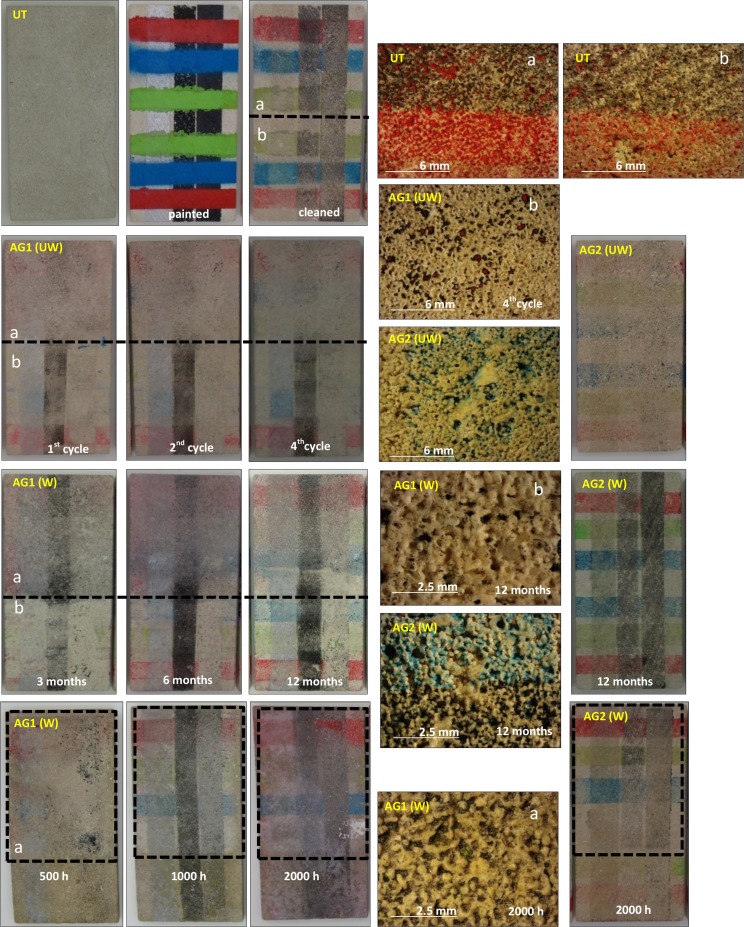
Pictures of graffiti removal on limestone slabs (150x75x10 mm). Untreated (UT) and protected with AG1 and AG2 anti-graffiti coatings, unweathered (UW) and natural (W) (3,6 and 12 months) and artificial weathered (W) (500, 1000 and 2000 h). Unweathered and natural weathered samples show two different cleaning procedures: top bottom (a), detergent and brush and half bottom (b), detergent and high pressurized water spray. The former is used on the marked (irradiated) area of the artificially weathered samples. Close-up images taken with a stereoscopy microscope.

**Fig 8:**
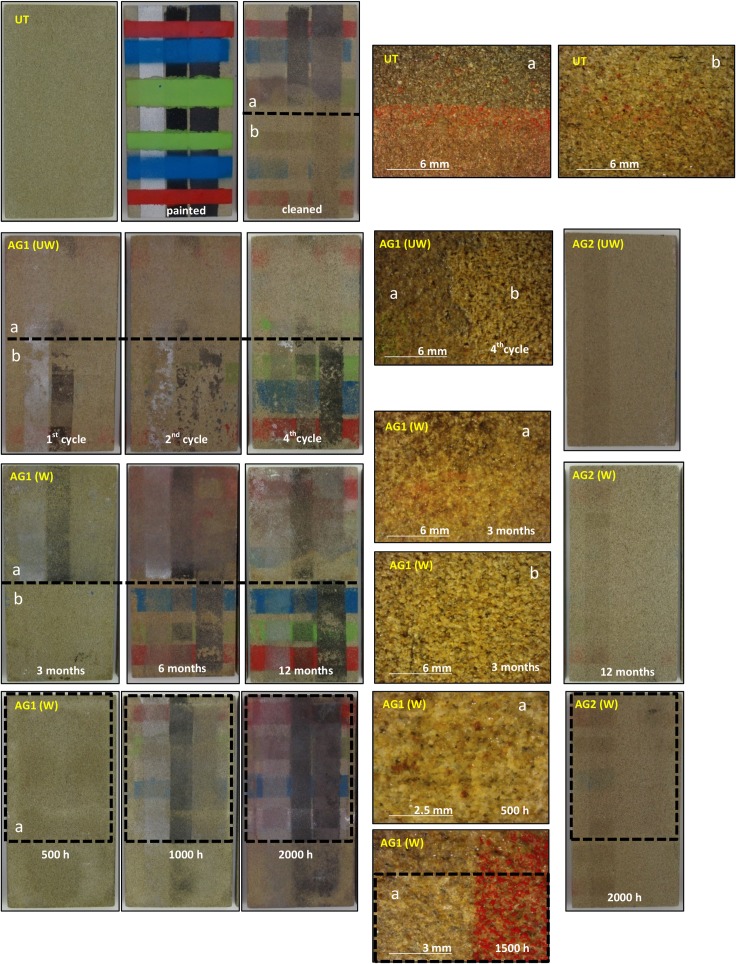
Pictures of graffiti removal on sandstone slabs (150x75x10 mm). Untreated (UT) and protected with AG1 and AG2 anti-graffiti coatings, unweathered (UW) and natural (W) (3,6 and 12 months) and artificial weathered (W) (500, 1000 and 2000 h). Unweathered and natural weathered samples show two different cleaning procedures: top bottom (a), detergent and brush and half bottom (b), detergent and high pressurized water spray. The former is used on the marked (irradiated) area of the artificially weathered samples Close-up images taken with a stereoscopy microscope.

**Fig 9:**
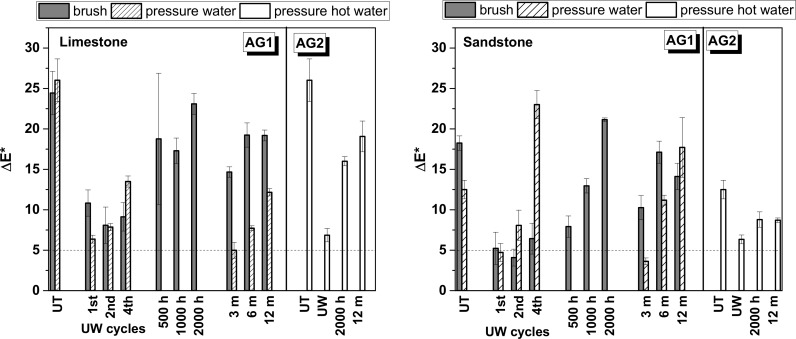
Overall colour changes on the surface of limestone and sandstone after graffiti removal. Untreated samples (UT), treated with AG1 and AG2 anti-graffiti coatings unweathered (UW) and artificially (500, 1000 and 200 h in a QUV chamber) and natural (3, 6 and 12 months of outdoor exposure) aged

Visual inspection of the stones showed (Figs 7 and 8) both unweathered anti-graffiti coatings facilitated graffiti removal in comparison with uncoated samples. On AG1-coated samples cleaning with brush was much more effective than pressurized water spray and colour stripes were hard to recognize, however paint residues remained on the surfaces which caused colour variations (ΔE*, Fig 9) of around 5 for sandstone, which would be deemed acceptable, and between 8 and 11 for limestone during the four painting/cleaning cycles. With pressurized water graffiti stripes were noticeable from the first cleaning episode, and overall colour changes (ΔE*) intensified with the increasing number of cycles, particularly on sandstone. This is mainly due, as revealed by stereographic imaging of sandstone (Fig 8, AG1(UW) a vs b), to the removal of the permanent anti-graffiti protection. On the contrary, on limestone the coating still remained on the surface after the fourth cleaning episode (Fig 7, AG1(UW) b) as a glossy finish was observed.

Colorimetric measurements after removal of paints and the sacrificial coating, AG2, were similar for both substrates, ΔE* = 6–7 (Fig 9, AG2-UW). Most of the paint was cleaned but residues of colour were retained in the porous system of the limestone whereas on sandstone almost complete colour removal was found apart from silver spray paint that left a shadow (oily residue) (Figs 7 and 8). Rivas et al. [35] reported the presence of a translucent film that modified the appearance of granite after removal of another silver spray paint.

In the literature graffiti removal is either evaluated with classification numbers based on pure visual inspection of the surfaces (from 0, complete removal to 5, not cleaned surfaces) or with overall colour variations (ΔE*). For the latter, various authors [15, 17, 36] have adopted the classification proposed by García and Malaga [12] to perceive colour changes in historic materials in which 10>ΔE*>5 are visible by human eye but acceptable and >10 are unacceptable. However, after combining both visual inspection and colour measurements in this study, it was considered that colour changes > 8 are not acceptable since graffiti colours are clearly recognized after graffiti removal.

**Fig 10:**
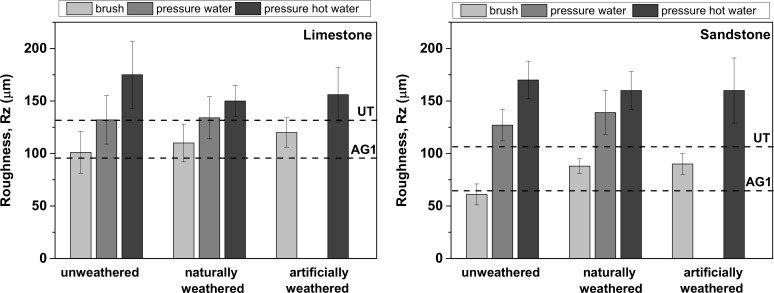
Roughness (Rz, μm) of the unweathered and weathered anti-graffiti coated surfaces after all episodes of graffiti removal. Reference values of the roughness of the samples without anti-graffiti coatings and with AG1 are displayed (not the ones for AG2 coated samples since this treatment does not modify the roughness of the stones).

Cleaning with detergent and brush did not modify the decrease on roughness (Rz) given by the AG1 coating to both stones (Fig 10). Carvalhão and Dionísio [36] have also reported that soft cleaning methods with either abrasives or chemical products are not damaging on calcareous stones. By contrast, pressurized water spray increased the roughness of the coated surfaces to values equal or higher than those of the substrates prior to the application of the anti-graffiti coating. Hot water pressure raised the roughness of the materials to a greater and significant extent (variation of Rz of approximately 50–60 μm in sandstone and 20–40 μm in limestone).

After weathering, acceptable results as monitored by colour change in terms of graffiti removal (ΔE* <8) were recorded on naturally weathered AG1 treated sandstone and limestone, for up to 3 months and 6 months respectively, after using pressurized water spray; on artificially aged (500 hours) AG1 sandstone cleaned with detergent and brush; and on AG2 coated sandstone both naturally and artificial weathered (Fig 9). This apparent effective cleaning is related to the fact that on those examples pressurized water spray removed not only sprays paints and pen but erode the surface of the stones with anti-graffiti coating included as revealed by the increase in roughness previously reported (Fig 10). When new painting and cleaning episodes were carried out (Figs 7 and 8, AG1 b) the adhesion of graffiti paint to the stones was favoured. As can be seen in stereomicroscope images in Figs 7 and 8, AG1 coating is removed from the surfaces by using pressurized water spray on aged limestone (Fig 7, AG1 (UW) b vs AG1 (W) b) and on sandstone both aged and unaged (Fig 8, AG1 (UW) b; AG1 (W) b 3 months)).

Cleaning with detergent and brush is a mild and effective procedure that neither increases the roughness of the stones (Fig 10) nor removes the anti-graffiti coatings from the surface (Figs 7 and 8, glossy finish on the stereomicroscope images AG1 4^th^ cycle) unless the treatment (AG1) has deteriorated. When this happens paints get smudged on the surface of the stones (Figs 7 and 8) and eventually the coating loses its adhesion (it is removed from the substrates as shown by the stereomicroscope images, AG1 (W) a 2000 h (Fig 7) and AG1 (W) a 1500 h (Fig 8).

## Conclusions

Prior to weathering both anti-graffiti coatings, the polyurethane with a perfluoropolyether backbone (AG1) and the crystalline micro wax (AG2) facilitate graffiti removal on Portland limestone and Woodkirk sandstone without relevant changes to their colorimetric coordinates and gloss. However, the polyurethane (AG1) adversely decreases the water vapour permeabilities of both stone types. On the AG1 coated samples, the combination of detergent and brush was the preferred cleaning procedure since pressurized water spray increases the roughness of the materials which favours graffiti adhesion in subsequent painting-cleaning episodes and even on sandstone the removal of the anti-graffiti protection.

Both anti-graffiti deteriorate under artificial and natural (in a temperate maritime climate) ageing trials:

- The surfaces coated with AG1 both yellowed and darkened. Furthermore, 2000 hours in the QUV chamber with UVB radiation were more aggressive to the coating than 1 year of outdoor exposure in temperate maritime conditions with the conditions in the chamber favouring loss of adhesion. However, under both artificial and natural weathering conditions the surfaces remained water-repellent.
- AG2 deteriorated equally under both types of weathering conditions. This led to a decrease in the yellow content and an increase in lightness of the surfaces, more relevant on the light coloured material, limestone, and to a reduction of their contact angles, on sandstone to below 90°.

As a result of these changes, cleaning efficiency of graffiti paints was lost on both permanent and anti-graffiti coatings.

These results show the suitability of running in parallel lab and field testing to assess the durability of anti-graffiti coatings since ageing phenomenon can vary from one test to another. For the conscientious use of these protective coatings in built heritage research still needs to be carried out regarding not only durability issues but mild graffiti removal procedures.
